# Serine/Threonine Protein Phosphatase 2A Regulates the Transport of Axonal Mitochondria

**DOI:** 10.3389/fncel.2022.852245

**Published:** 2022-03-18

**Authors:** Keunjung Heo, Himanish Basu, Amos Gutnick, Wei Wei, Evgeny Shlevkov, Thomas L. Schwarz

**Affiliations:** ^1^Kirby Neurobiology Center, Boston Children’s Hospital, Boston, MA, United States; ^2^Department of Neurobiology, Harvard Medical School, Boston, MA, United States

**Keywords:** mitochondrial transport, protein phosphatase 2A, indirubin, cantharidin, high-throughput screen

## Abstract

Microtubule-based transport provides mitochondria to distant regions of neurons and is essential for neuronal health. To identify compounds that increase mitochondrial motility, we screened 1,641 small-molecules in a high-throughput screening platform. Indirubin and cantharidin increased mitochondrial motility in rat cortical neurons. Cantharidin is known to inhibit protein phosphatase 2A (PP2A). We therefore tested two other inhibitors of PP2A: LB-100 and okadaic acid. LB-100 increased mitochondrial motility, but okadaic acid did not. To resolve this discrepancy, we knocked down expression of the catalytic subunit of PP2A (PP2CA). This long-term inhibition of PP2A more than doubled retrograde transport of axonal mitochondria, confirming the importance of PP2A as a regulator of mitochondrial motility and as the likely mediator of cantharidin’s effect.

## Introduction

The importance of mitochondria for ATP production, Ca^2+^ homeostasis, apoptosis, and cellular signaling has long been appreciated (Misgeld and Schwarz, 2017; Giacomello et al., 2020; Pfanner et al., 2021). The advent of fluorescent markers for live imaging revealed that mitochondria are highly dynamic organelles that move, divide, and fuse (Amiri and Hollenbeck, 2008; Liu et al., 2009; Wang and Schwarz, 2009). This dynamism regulates their distribution and enables them to support the energetic and metabolic needs of each portion of the cell. Because of the remarkable architecture of neurons, whose processes can extend over a meter from the cell body, mitochondrial motility is particularly critical in neurons (Schwarz, 2013; Misgeld and Schwarz, 2017). Indeed, their synapses, located in distal regions of axons and dendrites, can be the most energetically demanding regions of those cells (Chang et al., 2006). Therefore, providing healthy mitochondria to those extremities and replacing them during the lifetime of the organism is crucial to neuronal function. Perhaps in consequence, defects in mitochondrial motility have been reported in several neurodegenerative diseases and, in some cases, may contribute importantly to the pathology (Park et al., 2018).

The long-range transport of mitochondria in metazoans is accomplished primarily by the motors Kinesin-1 and Dynein operating on microtubule tracks (Saxton and Hollenbeck, 2012; Schwarz, 2013). The motors are coupled to the outer membrane of the mitochondrion by a protein complex that includes Miro (also called RhoT) and Trak (also called Milton) (Fransson et al., 2006; Glater et al., 2006). Perhaps reflecting the importance of an accurate and changing distribution of mitochondria as the energetic and metabolic needs of a cell change, this mechanism is subject to multiple regulatory pathways. Ca^2+^, for example, arrests mitochondria by binding to EF hands in Miro (Liu and Hajnoczky, 2009; MacAskill and Kittler, 2010). Increases in glucose availability also arrest mitochondria via the O-GlcNAcylation of Trak and subsequent anchoring to the actin cytoskeleton (Pekkurnaz et al., 2014; Basu et al., 2021). In addition, mitochondrial transport is altered by several kinases including PINK1, GSK3beta, JNK, and AuroraKinaseB (Morfini et al., 2002, 2006; Chada and Hollenbeck, 2003; Reed et al., 2006; Chen et al., 2007, 2010; Morel et al., 2010; Llorens-Martin et al., 2011; Ogawa et al., 2016; Shlevkov et al., 2016, 2019). During mitosis, multiple phosphorylations of Trak cause the motor proteins to be shed from the mitochondrial surface thereby detaching the mitochondria from the microtubule cytoskeleton and freeing the microtubules to engage in chromosome mechanics (Pack-Chung et al., 2007).

The list of modulatory pathways is not yet sufficient to explain many aspects of mitochondrial behavior in neurons, including why some mitochondria are stably anchored, why mitochondria are most likely to be anchored at synaptic sites, and what governs whether a mitochondrion moves to the + or − ends of the microtubules. Additional metabolic influences on mitochondrial dynamics are also likely to exist. To identify cellular pathways for mitochondrial regulation we have previously developed a compound screening platform called PATHS (Particle Analysis and Tracking for High-throughput Screening) based on high-content imaging of mitochondria in cultured neurons in a 96-well format (Carpenter et al., 2006; Bray and Carpenter, 2018; Shlevkov et al., 2019). This platform enables the user to track 10,000–40,000 mitochondria per well, distinguish stationary from motile mitochondria, and quantify the distance traveled by each mitochondrion during the imaging interval, typically 30 s per field. Because PATHS tracks very large numbers of the organelles, it offers highly accurate and reproducible quantification of mitochondrial motility in a given condition. Though PATHS does not offer the detailed description of motility available through kymography, the semi-automated analysis and greater reproducibility make it much more suitable for compound screening (Carpenter et al., 2006; Bray and Carpenter, 2018; Shlevkov et al., 2019).

Previously, we employed PATHS to identify three pathways of interest that enhance mitochondrial motility: AuroraKinaseB, Tripeptidylpeptidase 1 (TPP1), and the depolymerization of the actin cytoskeleton (Shlevkov et al., 2019). In the present study we sought to expand the list of pathways that regulate mitochondrial transport by screening for additional compounds that can enhance mitochondrial movement in neurons and by identifying their cellular targets. This screen uncovered two additional compounds that enhance mitochondrial motility and, together with short hairpin RNA (shRNA) data and kymography, pointed to protein phosphatase 2A (PP2A) as a regulator of mitochondrial motility in axons.

## Results

### Indirubin and Cantharidin Enhance Mitochondrial Motility

**FIGURE 1:**
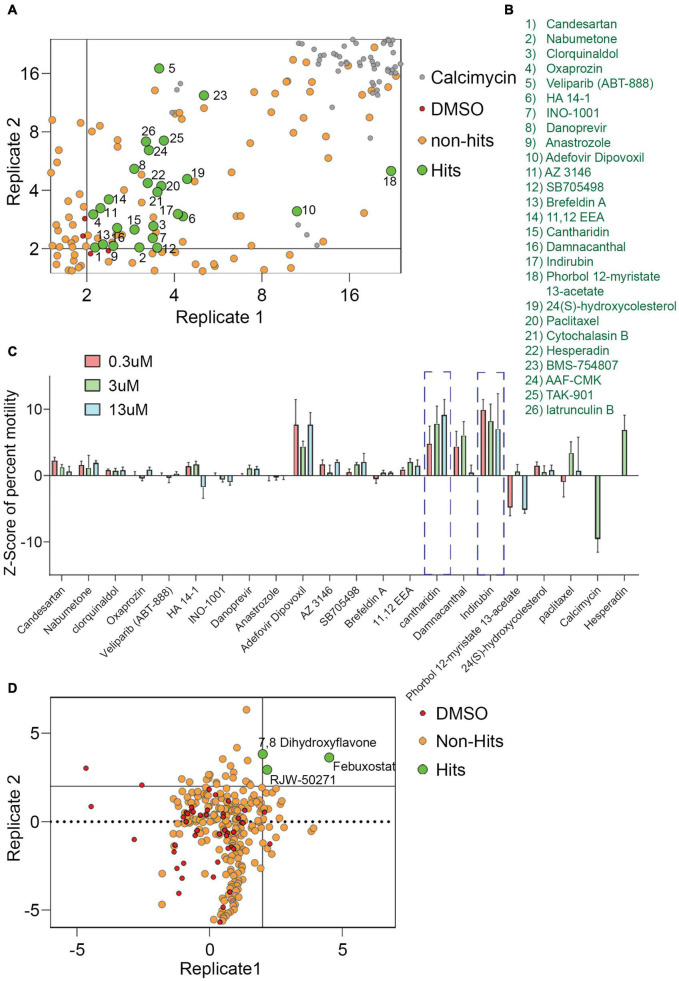
Screen of 1,641 compounds chosen from Selleck and Biomol libraries and 240 compounds chosen from the Mechanism of Action library. **(A,B)** From a previously published plot of *Z*-scores (KS of integrated distance) of 1,641 compounds, a selected region is shown that includes compounds of interest with *Z*-scores > 2. Each dot represents the *Z*-score of an individual compound from two replicate experiments. For the present study, a *Z*-score of 2 (*p* = 0.035) in both replicates was chosen as the criteria for identifying hits. Compounds that increased motility with a *Z*-score of more than 2 on both axes, i.e., hits, are green, numbered, and identified in **(B)**. Six compounds from this screen with *Z*-scores > 3 had previously been analyzed (Shlevkov et al., 2019) and are numbered 21–26. Compounds with no effect or that decreased motility were considered non-hits (tan). DMSO (red) and calcimycin (gray) controls are indicated. **(C)** *Z*-scores from retesting at 0.3, 3, and 13 μM of hit compounds 1–20. Calcimycin and Hesperadin were included as controls known to strongly decrease and increase mitochondrial movement. Out of the hits re-tested for confirmation, two hits, cantharidin and indirubin displayed a concentration dependent increase in mitochondrial movement and were followed up for further analysis. **(D)** Plot of *Z*-scores (median percent motile) of 240 compounds from the Mechanism of Action chemical library. As in **(A)**, each dot represents the *Z*-score of an individual compound from two replicates, each of which analyzed approximately 10,000 mitochondria in hundreds of axons. Compounds that increased motility with a *Z*-score > 2 in both replicates were considered as hits, while the remaining compounds were designated as non-hits. The 240-compound library revealed three compounds that significantly enhanced mitochondrial movement.

**FIGURE 2:**
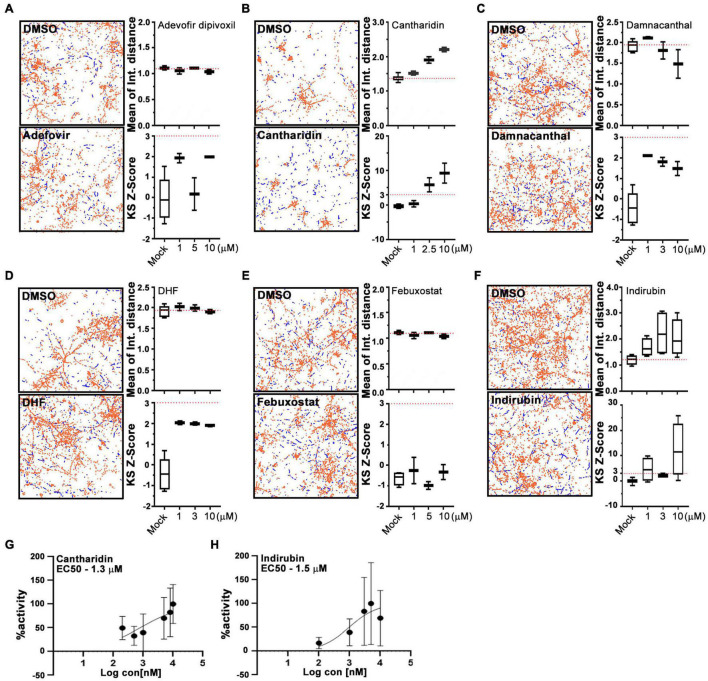
Cantharidin and indirubin enhance mitochondrial movement. **(A–F)** Representative fields with tracking of mitochondria as analyzed by the PATH algorithm in neurons treated with the indicated test compound and its DMSO control. Stationary mitochondria are red and the tracks of moving mitochondria are blue. From similar traces, and using three concentrations of each compound, the mean of the integrated distance traveled and the KS2 *Z*-score are shown to the right of each pair of traces. Dashed lines indicate the average value of integrated distance in control (DMSO) neurons and a *Z*-score of 3, the threshold used to select hits. **(G,H)** Dose-response curves of mitochondrial motility (integrated distance traveled) for cantharidin and indirubin plotted as the % maximum activity against the log of the concentration. Tukey’s box plots; error bars represent SD. All experiments were done in three replicates from different animals. In each of those replicates, four wells were imaged per condition, each well containing 30,000 neurons.

For a primary screen, rat hippocampal neurons were transfected with a mitochondrially targeted DsRed (MitoDsRed) on Day in Vitro (DIV) 6 or 7 and on DIV 8 or 9 test compounds were applied for 1 h before imaging mitochondrial movement. The images were analyzed with our previously published algorithm called PATHS that tracks more than 10,000 mitochondria per well in a 96-well plate format (Shlevkov et al., 2019). For the analysis, two parameters were used: the mean of the integrated distance traveled by mitochondria and the Kolmogorov–Smirnov (KS) *Z*-score of the integrated distance traveled. The integrated distance represents how far each individual mitochondrion traveled within the imaged field, summing the movement in all directions rather than only the net displacement from its starting position (Shlevkov et al., 2019). The KS *Z*-score of the integrated distance represents the statistical significance of the integrated distance value in a compound-treated condition relative to the mock-treated condition. The efficacy of the screen was confirmed by comparing the vehicle (DMSO) to calcimycin, which arrests mitochondrial movement by elevating cytosolic Ca^2+^ (Supplementary Figure 1). We previously screened 1,641 compounds from the Selleck Bioactive collection and Biomol collection in this manner. Six enhancers of mitochondrial motility were found with *Z*-scores greater than 3 in each of two replicates: two actin depolymerizers (Latrunculin B and Cytochalasin B), three kinase inhibitors that acted through Aurora Kinase B (BMS-754807, Hesperadin, and TAK-901), and one peptidase inhibitor (AAF-CMK) (Shlevkov et al., 2019; Figures 1A,B,D). To discover additional enhancers, we returned to the output of that screen and selected 20 compounds that had a *Z*-score greater than 2. These compounds (Figures 1B,C) were rescreened at three concentrations: 0.3, 3, and 13 μM (Figure 1C) and from that rescreening 4 promising hits were brought forward for further investigation (indirubin, cantharidin, adefovir dipovoxil, and damnacanthal). We also used the screening platform to test in duplicate 240 compounds from a library of compounds with known mechanisms of action (Supplementary Table 1). We identified three, febuxostat, 7,8-Dihydroxyflavone hydrate (DHF) and RWJ-50271, as having *Z*-scores greater than 2 in both replicates (Figure 1D). Because RWJ-50271 is a frequently encountered false-positive, only febuxostat and DHF were brought forward for further testing. The six selected compounds were then rescreened after 1 h treatment with doses ranging from 1 to 10 μM (Figures 2A–F). In this rescreening, only indirubin and cantharidin had a reasonable dose/response relationship for enhancing the movement of the neuronal mitochondria (Figures 2A–F). To further examine their efficacy, we established their dose-response relationships over a broader range of concentrations (Figures 2G,H). The resulting estimates of their effective concentration (EC50) values were 1.3 μM for cantharidin and 1.5 μM for indirubin and these two compounds were selected for further investigation.

### The Effects of Indirubin on Mitochondrial Transport

**FIGURE 3:**
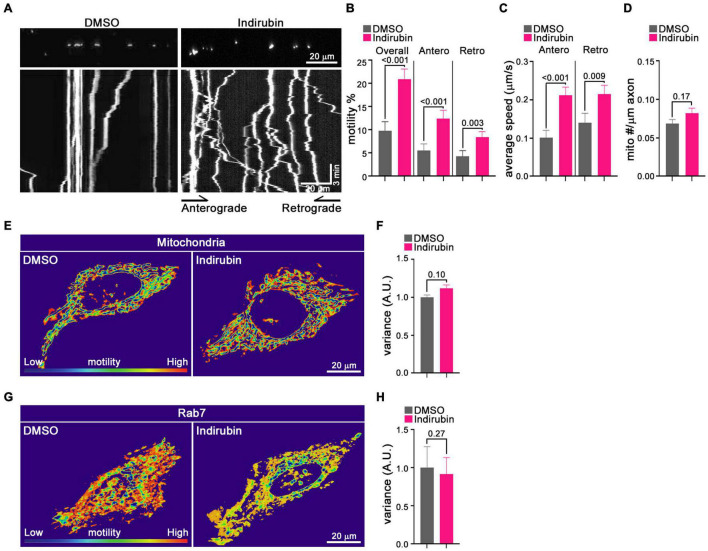
Indirubin increases movement of axonal mitochondria. **(A–D)** Kymograph analysis of mitochondrial motility in rat cortical neurons treated with DMSO or indirubin. **(A)** Representative images of mitochondria in DMSO and indirubin treated axons. Top panels show images of multiple mitochondria in representative axon segments. Bottom panels show the corresponding kymographs generated from 3-min timelapse imaging of the axon segments. Below each image are kymographs built from 3 min time-lapse movies. The *x*-axis corresponds to the position of the mitochondrion in the axon shown above and on the *y*-axis is time proceeds downward. Anterograde movement is to the right. The percent of mitochondria that were motile **(B)**, their average velocity in either direction **(C)**, and mitochondrial density along the axon **(D)** were derived from kymographs as in **(A)**. Statistical comparisons to the DMSO-treated controls are from nonparametric Mann–Whitney tests. All experiments were done in three independent cultures. A total of 16 axon segments (109 mitochondria) for DMSO and 19 axon segments (156 mitochondria) for indirubin treated neurons were analyzed. Each axon segment was selected from a separate neuron. Bars represent the average motility of all mitochondria analyzed for each condition. Error bars represent SEM. **(E–H)** The movement of mitochondria and Rab7 in Hela cells treated with DMSO and indirubin. QuoVadoPro generates heatmaps based on the pixel variance of fluorescently labeled mitochondria **(E)** or Rab7 **(G)**. Scale bar, 20 μm. The variance score was normalized to DMSO **(F,H)**. The statistical comparisons were by nonparametric Mann–Whitney test **(F)** and student *t*-test **(H)**. Each bar represents an average of 20–30 cells over three replicates, and error bars indicate SEM.

Although the primary screen quantified mitochondrial motility, it did not discriminate between anterograde and retrograde movements and, though biased toward analysis of axons, contained some dendritic mitochondria as well. Therefore, to characterize further indirubin’s effect on axonal mitochondria, we used the more conventional method of kymography on individual identified axons. Mitochondria in cortical neurons were labeled by transfection with MitoDsRed on DIV 6 or 7 and imaged 2 days post-transfection. Prior to imaging, neurons were incubated with DMSO (control) or 10 μM indirubin for 1 h. To quantify axonal mitochondrial movement, a 70–115 μm length of an individual axon was imaged for 3 min. Movement was analyzed using the Kymolyzer algorithm (Basu and Schwarz, 2020) to extract the density of mitochondria per μm of axon, the percent of mitochondria that were motile during the imaging period in each direction, and their velocities. The percent motile was 9.9% in control (DMSO) axons but increased to 20.9% with indirubin (Figures 3A,B). The enhancement of motility was evident in both directions: indirubin increased the anterograde percent motile from 5.5 to 12.5% and retrograde from 4.3 to 8.4% (Figure 3B). The average velocity of mitochondria was also increased for both anterograde from 0.10 to 0.21% and retrograde from 0.14 to 0.21% in indirubin-treated neurons (Figure 3C). Mitochondrial density along the axon was not appreciably altered (Figure 3D).

To determine whether indirubin influenced mitochondrial movement in non-neuronal cells, we applied a customized algorithm called QuoVadoPro (Basu and Schwarz, 2020) to HeLa cells transfected with MitoDsRed. This algorithm is well-suited for analysis of cells in which mitochondria can be in a reticulum rather than discrete organelles, and where microtubules are not arrayed in parallel like in an axon. The algorithm emphasizes progressive movement over jiggle and the pixel variance of the fluorescently tagged mitochondria serves as a proxy for motility. Compared with control Hela cells, the pixel variance of MitoDsRed from 3 min time-lapse movies was at most slightly increased (11%, *p* = 0.10) after the indirubin treatment (Figures 3E,F). We also examined the effects of indirubin on other organelle movement in Hela cells. The pixel variance of mCherry-Rab7, which labels late endosome/lysosomes, was not increased in indirubin-treated cells (Figures 3G,H). Thus, indirubin was most effective in neurons and may be selectively enhancing mitochondrial transport.

### Mitochondrial Movement Was Not Enhanced by Inhibitors of Glycogen Synthase Kinase 3 Beta and Cyclin-Dependent Kinases

Indirubin inhibits glycogen synthase kinase 3 beta (GSK-3β) and cyclin-dependent kinases (CDKs) including CDK1, CDK2, CDK4, and CDK5 (Hoessel et al., 1999). In addition, an indirubin derivative, indirubin E804, that acts as type I CDK16 inhibitor, influences neurite outgrowth and vesicular transport within axons (Dixon-Clarke et al., 2017). To probe whether any of these kinases are the cellular target responsible for the action of indirubin on mitochondrial motility, we identified additional inhibitors of these kinases and tested them at three concentrations. GSK-3β was of particular interest because it has previously been shown to inhibit mitochondrial transport when activated (Yang et al., 2017). GSK-3β inhibitors can be categorized as either ATP-competitive or non-ATP-competitive inhibitors (Orena et al., 2000; Eldar-Finkelman and Martinez, 2011; Kalra et al., 2017; Levin et al., 2017; Dan et al., 2020). We selected four ATP-competitive inhibitors (AR-A014418, indirubin-3′-monoxime, 6-BIO, and TWS119) and two ATP-noncompetitive inhibitors (tideglusib and Li+) (Phiel and Klein, 2001; Noble et al., 2005; Tolosa et al., 2014) to test in our assay, but none of these resembled indirubin and enhanced mitochondrial movement; some caused a modest inhibition (Supplementary Figure 2A). We also tested compounds annotated as selective inhibitors of CDK1, CDK2, CDK4, and CDK5. Compared with the mock condition, mitochondrial motility was decreased after the treatment with AUZ (K03861), a selective CDK2 inhibitor (Tang et al., 2018; Supplementary Figure 2B). R547, roscovitine, and SU9516, three inhibitors reported to parallel indirubin in their inhibition of CDKs were also tested. R547 is an ATP-competitive inhibitor for CDK1, CDK 2 and CDK4 (DePinto et al., 2006), roscovitine, is an ATP-competitive inhibitor for CDK1, CDK2, and CDK5 that releases cytochrome c from mitochondria and induces apoptosis (De Azevedo et al., 1997; Edamatsu et al., 2000). SU9516 has been described as a potent selective inhibitor of CDK1, CDK2 and a weak inhibitor for CDK4 (Lane et al., 2001). None of these CDK inhibitors, however, enhanced mitochondrial motility in our assay and indeed several reduced it (Supplementary Figure 2B). This was also the case with dabrafenib and rebastinib, CDK16 inhibitors (Dixon-Clarke et al., 2017; Supplementary Figure 2B). Five compounds were re-examined over a broader concentration range, but still failed to mimic Indirubin’s ability to enhance mitochondrial transport in a dose-response manner (Supplementary Figures 2C–G). The failure of these kinase inhibitors to mimic indirubin appears rule out these CDKs and GSK-3β as the cellular targets responsible for the enhanced mitochondrial transport; we have not been able to associate this action of indirubin with any of its annotated target kinases.

### Protein Phosphatase 2A Inhibition Can Enhance Mitochondrial Motility

**FIGURE 4:**
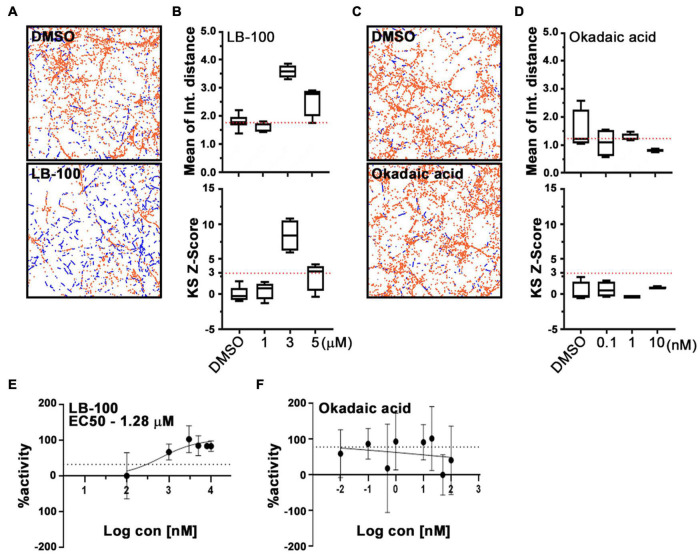
LB-100, but not okadaic acid, enhances mitochondrial movement in cortical neurons. Representative fields **(A,C)** and quantification **(B,D)** of mitochondrial movement in neurons treated with LB-100 or okadaic acid and their DMSO controls, as analyzed with PATHS, as in Figure 2, stationary mitochondria are red and the tracks of moving mitochondria are blue. The mean of integrated distance and KS2 *Z*-scores of mean of integrated distance were quantified, and dotted lines indicate the average mean integrated distance in DMSO and a *Z*-score of 3. **(E,F)** Dose-response curves for LB-100 and okadaic acid plotted as a fraction of maximum activity. Tukey’s box plot was used for all data, and the error bars represent SD. Experiments were done at least in three independent cultures and the mitochondria were imaged in four individual fields per condition in each culture, each field containing dozens of axons.

Cantharidin is a potent inhibitor of the serine/threonine protein phosphatases types 1 (PP1) and 2A (PP2A) and a weak inhibitor of type 2B (PP2B) (Honkanen, 1993). The reported half-maximal inhibitory concentration (IC50) values for cantharidin on these targets are ∼0.16 μM for PP2A and ∼1.70 μM for PP1, and >500-fold higher IC50 value for PP2B than PP1 in PC12 cells (Li and Casida, 1992; Honkanen, 1993). To determine whether any of these phosphatases are responsible for the effect of cantharidin on mitochondria motility, we examined cortical neurons treated with two other protein phosphatase inhibitors, LB-100, and okadaic acid (Figure 4). LB-100 inhibits the activity of PP2A with an IC50 value of ∼0.4 μM and for PP1 at ∼80 μM in the human glioblastoma multiforme cell line U87MG (Lu et al., 2009). In contrast, okadaic acid potently inhibits PP2A at ∼0.04 nM and PP1 at 10–200-fold higher concentrations than PP2A in PC12 cells (Honkanen, 1993; Lu et al., 2009; Hu et al., 2017). IC50 values for these compounds have not been determined in rat primary neurons. To explore the effectiveness of these compounds on mitochondrial motility, rat cortical neurons on DIV 8 or 9 were treated with the compounds for 1 h prior to imaging using PATHS to establish a dose-response curve. Compared with control cells, mitochondria motility was significantly increased in neurons treated with 3 μM LB-100 (Figures 4A,B). From the sigmoidal dose-response curve, an IC50 value for LB-100 to enhance mitochondrial motility was at ∼1.28 μM (Figure 4E), implicating PP2A as the most likely target. Unlike LB-100, however, okadaic acid did not enhance mitochondrial motility and no clear dose-response relationship could be established over a broad range of concentrations (Figures 4C,D,F).

**FIGURE 5:**
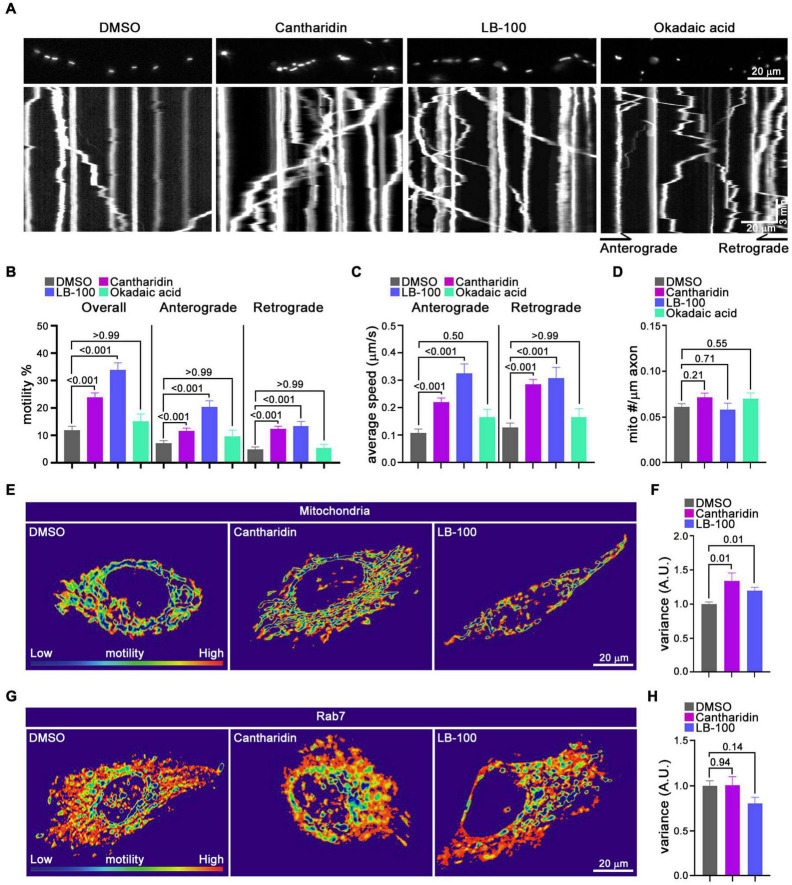
Cantharidin and LB-100 increase both anterograde and retrograde mitochondrial transport. **(A–D)** Kymograph analysis of mitochondrial transport in axons of rat cortical neurons treated with DMSO, cantharidin, LB-100 and okadaic acid. **(A)** Representative images of axons with labeled mitochondria above kymographs derived from movies of those axons. The percent of mitochondria that were motile **(B)**, their average velocity in either direction **(C)**, and mitochondrial density along the axon **(D)** were derived from kymographs as in **(A)**. Statistical comparisons to the DMSO control were by nonparametric Kruskal–Wallis **(B,C)**, and one-way ANOVA **(D)**. The movement of mitochondria **(E,F)** or Rab7-tagged endosomes **(G,H)** in Hela cells as illustrated with heat maps generated in QuoVadoPro **(E,G)** and quantification of their motility **(F,H)**. The number of cells quantified for mitochondria: *n* = 20 (DMSO), *n* = 18 (Cantharidin), *n* = 10 (LB-100); for Rab7: *n* = 23 (DMSO), *n* = 15 (cantharidin), *n* = 12 (LB-100). The variance score was normalized to DMSO. Statistical comparisons are with nonparametric Kruskal–Wallis tests **(F)** and one-way ANOVA test **(H)**. All experiments were done in three independent cultures. A total of 35 axon segments (231 mitochondria) for DMSO, 40 axon segments (326 mitochondria) for cantharidin, 19 axon segments (152 mitochondria) for LB-100, and 11 axon segments (70 mitochondria) for okadaic acid treated neurons were analyzed. Each axon segment was selected from a separate neuron. Bars represent the average motility of all mitochondria analyzed for each condition. Error bars represent SEM.

The influence of cantharidin, LB-100 and okadaic acid on mitochondria was also examined by kymography in the axons of cortical neurons transfected with MitoDsRed (Figure 5). The percent of mitochondria undergoing anterograde transport increased upon treatment with either 10 μM cantharidin (166% of control) or 3 μM LB-100 (291% of control). Retrograde transport was also increased by cantharidin (249%) and LB-100 (271%) (Figures 5A,B). Both compounds also increased the velocity in both directions and the density of mitochondria (Figures 5C,D). Consistent with the data in Figure 4, however, okadaic acid did not consistently increase mitochondrial transport in either direction (Figures 5A–C), nor did it detectably alter mitochondrial density (Figure 5D). To examine the effects of cantharidin and LB-100 in non-neuronal cells, we again used the QuoVadoPro algorithm in HeLa cells, as in Figure 3. Both compounds increased mitochondrial motility (Figures 5E,F) but not the motility of mCherry-Rab7-tagged endosomes (Figures 5G,H). Thus, cantharidin and LB-100 will enhance mitochondrial transport in both neurons and HeLa cells and this action is at least in part selective for mitochondria.

### Knockdown of the Protein Phosphatase 2A Catalytic Subunit Increases Transport of Axonal Mitochondria

**FIGURE 6:**
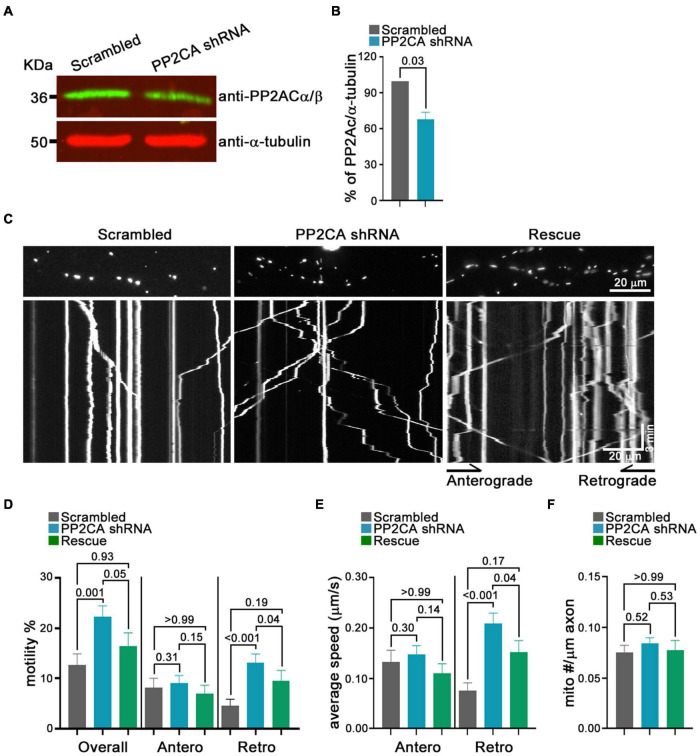
Knockdown of the PP2A catalytic subunit increases retrograde transport of axonal mitochondria. **(A)** Representative Western blot of neurons transfected with shRNA against PP2AC or a scrambled shRNA and probed with anti-PP2Ac α/β (green) and anti-α-tubulin (red) demonstrates efficacy of the shRNA. **(B)** Quantification of PP2CA normalized to α-tubulin from Western blots as in **(A)**. *N* = 3, error bars represent mean±SEM. **(C–F)** Kymograph analysis of mitochondrial transport in cortical axons expressing scrambled shRNA, PP2A shRNA, and an shRNA-resistant PP2A rescue construct. Representative kymograph images of mitochondria **(C)** and quantification of the percent of mitochondria that were motile, overall or in either anterograde or retrograde directions **(D)**, their average velocity **(E)**, and the density of mitochondria in axons for each condition **(F)**. Statistical comparisons to the scrambled shRNA are from nonparametric Kruskal–Wallis tests. All experiments were done in three independent cultures. A total of 19 axon segments (135 mitochondria) for scrambled shRNA, 25 axon segments (210 mitochondria) for PP2A shRNA, and 19 axon segments (137 mitochondria) for shRNA-resistant PP2A rescue neurons were analyzed. Each axon segment was selected from a separate neuron. Bars represent the average motility of all mitochondria analyzed for each condition. Error bars represent SEM.

**FIGURE 7:**
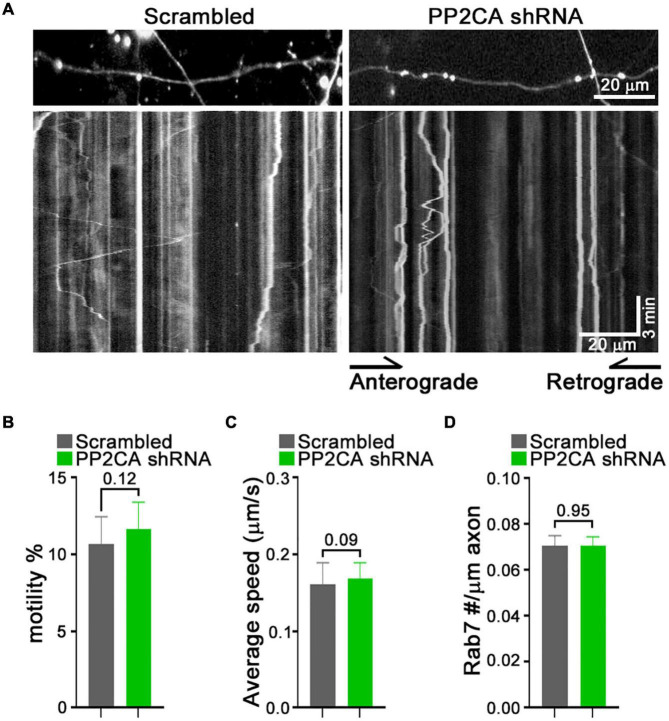
Knockdown of PP2A catalytic subunit does not detectably alter rab7 transport. **(A–D)** Kymograph analysis of mCherry-Rab7-tagged late endosomes and lysosomes in neurons expressing a scrambled shRNA or PP2A shRNA. Representative kymograph images **(A)** and quantification of the percent of motile rab7-tagged organelles **(B)**, their average velocity **(C)**, and their density along the axons **(D)**. Statistical analysis was by nonparametric Mann–Whitney test **(B,C)**, and student *t*-test **(D)**. All experiments were done in three independent cultures. A total of 24 axon segments (177 mitochondria) for scrambled shRNA, and 25 axon segments (184 mitochondria) for PP2A shRNA neurons were analyzed. Each axon segment was selected from a separate neuron. Bars represent the average motility of all mitochondria analyzed for each condition. Error bars represent SEM.

From this pharmacological analysis, PP2A emerged as a likely candidate to be the relevant cellular target of cantharidin and LB-100, although the lack of efficacy of okadaic acid was a discordant finding. To test directly whether PP2A was an inhibitor of mitochondrial transport, we used shRNA to knockdown the catalytic subunit of PP2A (PP2CA) in neurons. We established the knockdown efficiency of PP2CA shRNA by introducing them by lentiviral transduction into cortical neurons. After 48–72 h, PP2CA levels were assessed by Western blot (Figure 6A) and found to be reduced by 32% relative to neurons transduced with a scrambled shRNAs (Figures 6A,B). When mitochondrial transport was then assayed by kymography in cortical axons, the PP2CA shRNA increased the percent of motile mitochondria to 22.2% compared to 12.7% with the control shRNA (Figures 6C,D). Most prominently, it was the percent of retrograde transport that increased from 4.5% (control) to 13.1% (PP2A shRNA), with a more than twofold increase in the average velocity of retrograde transport as well (Figures 6D,E). The effects on anterograde transport were more modest and lacked compelling statistical significance (*p* = 0.3) (Figures 6D,E). The effects of the shRNA on retrograde motility were not due to off-target effects; expression of a PP2CA that was resistant to the shRNA restored mitochondrial movement to levels in control axons (Figures 6D,E). As observed with pharmacological inhibition, mitochondrial number per axonal length was not detectably altered by knockdown of PP2CA (Figure 6F). We examined the selectivity of PP2CA knockdown for mitochondrial transport by applying kymography to mCherry-Rab7 tagged endosomes. Compared with control axons, neither the average speed, nor the number of Rab7 organelles were appreciably altered in PP2CA-knockdown axons, though there may have been a modest increase in the percent that were motile from 10.7 to 11.7% (*p* = 0.12) (Figures 7A–D). Thus, the shRNA data confirm the finding implied by the pharmacology of cantharidin and LB-100 that PP2A is restraining mitochondrial motility in cortical axons (Figures 6D–F).

## Discussion

Protein phosphorylation is important for regulating many cellular mechanisms. Using a high-throughput screening platform, we previously found that Aurora Kinase B (AurKB) regulates mitochondrial movement (Shlevkov et al., 2019); inhibition of AurKB enhanced mitochondrial movement. By investigating additional small-molecule compounds at multiple concentrations two compounds, indirubin and cantharidin, that also enhance axonal transport of mitochondria in both anterograde and retrograde directions and increase mitochondrial velocities. We were unable, however, to identify a cellular target for indirubin responsible for this action. Indirubin inhibits GSK3β and multiple CDKs (Leclerc et al., 2001; Marko et al., 2001; Eldar-Finkelman and Martinez, 2011; Dixon-Clarke et al., 2017; Levin et al., 2017), but other inhibitors of these kinases did not reproduce the efficacy of indirubin (Supplementary Figure 2) in our assays. Mimicking indirubin might require the inhibition of a broad spectrum of kinases that was not matched any of the individual inhibitors we tested (Davis et al., 2011). Alternatively, the cellular target that mediates the effect on mitochondrial movement may be an as yet unknown target of indirubin.

The action of cantharidin on axonal mitochondria was mimicked by the phosphatase inhibitor LB-100. The IC50 values we obtained in rat cortical neurons were ∼1.3 μM for cantharidin and ∼1.28 μM for LB-100 (Figures 2, 4). These values are in better agreement with the reported values for PP2A inhibition in cell lines (0.16 and 0.4 μM) than those for other phosphatases (Li and Casida, 1992; Honkanen, 1993; Lu et al., 2009). We confirmed that PP2A inhibits mitochondrial motility by using shRNA against its catalytic subunit (Figures 6, 7). The specifics of how neuronal mitochondria responded to the pharmacological inhibition and knockdown by shRNA differed in some regards; in particular the shRNA, unlike cantharidin or LB-100, had a greater effect on retrograde than anterograde transport. This difference is likely to arise from the distinction between chronic knockdown by shRNA and acute (1–2 h) treatment with the drugs.

We do not have a clear understanding of why another protein phosphatase inhibitor, okadaic acid, did not enhance mitochondrial motility. Okadaic acid inhibits PP2A with an IC50 ∼0.04 nM and PP1 at 10–100-fold higher concentrations (Honkanen, 1993; Kamat et al., 2014). Nevertheless, across a broad range of concentrations okadaic acid did not increase axonal transport of mitochondria. PP2A, however, is a complex enzyme whose trimeric subunit composition is so varied that at least 96 different holoenzymes may occur and whose activity can be further regulated by the non-canonical subunits α4 and TIRPL1. This complexity, as well as post-translational modifications, creates diverse substrate specificities and activities of the assembled enzyme. It is possible that some modes of PP2A activation differ in their sensitivity to okadaic acid (Smetana and Zanchin, 2007; Sents et al., 2013). The chemical structures of cantharidin and its related compound LB-100 are quite distinct from that of okadaic acid (Stewart et al., 2007). Recent papers have noted that PP5C possesses a catalytic site very similar to that of PP2A and both PP5C and PP2A could be inhibited by LB-100 (Hamilton et al., 2018; D’Arcy et al., 2019). Thus some of the efficacy of cantharidin and LB-100 may be due to the simultaneous inhibition of both phosphatases. This may also in part explain why knockdown of PP2A did not perfectly match the effects of cantharidin and future studies should examine a role for PP5C in regulating mitochondrial motility. We additionally examined mitochondrial movement in neurons after treatment with a bioavailable PP2A activator called DT-061. However, the compound did not detectably alter mitochondrial motility (data not shown). DT-061, however, is known to increase PP2A activity on only on some substrates and not others (Leonard et al., 2020), which may explain its lack of efficacy in our assay.

The identification of PP2A as a regulator of mitochondrial axonal transport adds to a growing body of evidence for the importance of phosphorylations in regulating mitochondrial motility. Many of the key elements of the transport apparatus are phosphoproteins. Milton/Trak has at least 28 known phosphorylation sites (Pack-Chung et al., 2007) and Miro/RhoT, kinesin, dynein and dynactin are also phosphorylated (Olsen et al., 2010; Wang et al., 2011; Chung et al., 2016). Microtubules and their associated proteins, including tau, are also heavily phosphorylated (Sontag et al., 1999; Akhmanova and Steinmetz, 2015; Goodson and Jonasson, 2018). Indeed, PP2A directly binds to tau and regulates its phosphorylation state (Cheng and Bai, 2018; Taleski and Sontag, 2018). Any of these proteins, or all of them, may be the protein targets of PP2A that account for the ability of cantharidin and LB-100 to increase axonal transport of mitochondria.

Regulation of mitochondrial transport is crucial to neuronal function and mitochondrial transport defects are linked with neurodegenerative diseases, including amyotrophic lateral sclerosis (ALS) and Parkinson’s disease (Sasaki et al., 2005; Bilsland et al., 2010; Schwarz, 2013). Mutations in either cargo binding domain of Kif5A or the dynein activator dynactin disrupt mitochondrial transport and are associated with ALS (Morfini et al., 2009; Brenner et al., 2018; Nicolas et al., 2018). ALS-causing SOD1 mutations also impair mitochondrial transport in axons (Bilsland et al., 2010). Compound screening, as undertaken in this study, may identify cellular targets important for mitochondrial transport. Further dissection of the role of protein phosphorylation and dephosphorylation in this process may therefore offer new strategies for ameliorating the pathogenesis of neurodegeneration.

## Materials and Methods

### DNA Constructs

*pDsRed2-Mito* (MitoDsRed) was a gift of G. Hajnoczky (Thomas Jefferson University, Philadelphia, PA, United States). For shRNA constructs, *pLKO.1-non-target shRNAs* and *pLKO.1-PP2Ac shRNAs* (TRCN0000002484) were obtained from Sigma-Aldrich (St. Louis, MI, United States). The EGFP fragment was amplified by PCR from mEGFP.N1 and cloned into *pLKO.1-non-target-shRNA* (Scrambled) and *pLKO.1-hPP2A-shRNA* (PP2A shRNA) to generate *pLKO.1-non-target-EGFP* and *pLKO.1-hPP2A-shRNA-EGFP*. *pLKO.1-non-target-EGFP* and *pLKO.1-hPP2A-shRNA-EGFP* expressing lentivirus was produced at Boston Children’s Hospital (BCH) Viral Core. *PP2A-shRNA-Resistant-BFP* was generated using Q5 Hot Start High-Fidelity site-directed mutagenesis kit (New England Biolabs, #M0494, Ipswich, MA, United States). *mCherry-Rab7* (#55127) and *mEGFP-N1* (#54767) were obtained from Addgene.

### Cell Culture

Hela cells were maintained in DMEM, high glucose, GlutaMAX™ (ThermoFisher Scientific, Waltham, MA, United States) supplemented with 10% FBS (Atlanta Biological, Minneapolis, MN, United States), 1% penicillin/streptomycin (ThermoFisher Scientific) in 5% CO2 at 37°C. To transfect the cells, Lipofectamine2000 (ThermoFisher Scientific) and TransIT-LT1 (Mirus, Madison, WI, United States) were used. Primary cortical neurons isolated from E18 rat embryos (Charles River) were seeded on 20 μg/mL poly-D-lysine (Sigma-Aldrich), and 3.5 μg/mL laminin (ThermoFisher Scientific) coated plates. Neurons were maintained in Neurobasal Medium (Life Technologies, Carlsbad, CA, United States) supplemented with 2% B27 (GIBCO, Carlsbad, CA, United States), 100 U/mL penicillin/streptomycin (ThermoFisher Scientific), in 5% CO2 at 37°C. For spot-groove cultures, cortical neurons were plated on 20 μg/mL poly-D-lysine (Sigma-Aldrich), and 3.5 μg/mL laminin (ThermoFisher Scientific) coated μ-24-well plates with polymer coverslip bottoms (ibidi, Fitchburg, WI, United States). Each plate was scratched with a CAMP-PR pin rake (Tyler Research Corporation, Edmonton, AB, Canada) to promote separate growth of neuronal cell bodies and projecting axons in the plate. For this culture, approximately 1 × 10^4^ neuronal cells per well were seed in the center of the 24-well plates. After 1 h, more media was added and cells were maintained in those conditions until transfection.

### Western Blot

Rat cortical neurons were washed once with ice-cold PBS and homogenized in cold NP-40 buffer (1% Nonidet P-40, 50 μM Tris–HCl, 5 mM EDTA, and protease inhibitor cocktail). The primary antibodies were anti-PP2A-Cα/β (1:1,000; Santa Cruz Biotechnology), anti-α-tubulin (1:1,000; Sigma-Aldrich). Western blots are done for three independent experiments.

### Time-Lapse Live Imaging of Mitochondrial Motility

MitoDsRed was introduced into Hela Cells or cortical neurons at DIV6-7 using Lipofectamine2000 transfection reagent (ThermoFisher Scientific). Both Hela cells and neurons were imaged 2 days post-transfection. Prior to imaging, neurons were incubated with the indicated compound in either regular growth media or phenol-free Hibernate E media (BrainBits). The MitoDsRed was imaged 1–3 h post incubation with compounds.

#### For Analysis of Axonal Mitochondrial Transport *via* Kymographs

The neuronal cultures were imaged on a Nikon Ti-Eclipse equipped with an environmental chamber that was supplied with humidified 5% CO2 and maintained at 37°C. Axon segments were imaged using Plan Apo 20X (NA0.75) and Plan Fluor 60X (NA1.4) objective. For each time-lapse images, a selected axon segment was imaged for 3 min at frame rate of 2 Hz.

#### For Mitochondrial Motility Analysis on a Large Scale

Approximately 30,000 cortical neurons per well were plated for live-imaging in a 96-well plate using 20× objective with 2 × 2 binning using ArrayScan XTI imaging platform (ThermoFisher Scientific). Thirty frames were imaged per field with a maximum speed 10 Hz, and more than four fields were acquired per well. A total of 10 μM DMSO and 10 μM calcimycin were used for negative and positive controls. Individual neurons were not distinguishable from the MitoDsRed signals, but each field imaged included the axons of many dozens of neurons so that the movement of 10,000 mitochondria per well could be analyzed.

### Data Analysis

Hela cells and cortical neurons were imaged live for 3 min on a TiEclipse microscope. The analysis of the mitochondrial variance score in Hela cells and axonal mitochondrial transport in cortical axons were analyzed with the custom scripted QuoVadoPro, and Kymolyzer algorithms (Basu et al., 2020; Basu and Schwarz, 2020). For analysis of large-scale images, time-lapse images obtained from the ArrayScan XTI imaging platform were analyzed using CellProfiler pipelines (Carpenter et al., 2006; Jaqaman et al., 2008) and PATH algorithms in MATLAB (MathWorks, Natick, MA, United States). From the algorithms, stationary and motile mitochondria were distinguished, and then the movement made by each motile mitochondrion was analyzed per field to derive the mean of integrated distance. The KS *Z*-score was used to determine the statistical significance. For dose-response curves, each concentration was converted to log concentration (nM). The mean of integrated distance value was converted to the percent maximum activity by determining the low and high values within the individual data set and internally normalized. For plotting, the log (agonist) versus normalized response-variable slope were used in Prism (GraphPad, San Diego, CA, United States).

### Statistical Analysis

For high-content data analysis, MATLAB was used for *Z*-score measurements, using the formula Z = (x − μ)/σ for each parameter. Mean±SEM. for each parameter were obtained from samples described in Figures 2, 4 and Supplementary Figures 2, 3. For a low-content data analysis, the statistical analyses were done using Prism (GraphPad). For a two-sample comparison, the normal distribution of the mean value was tested using D’Agostino and Pearson omnibus normality test and the variance was tested using *F*-test. If the value is normally distributed, the Student’s *t*-test was used for statistical analysis. Otherwise, nonparametric Mann–Whitney test was used. For multi-sample comparison, the normal distribution of the mean value was tested using D’Agostino and Pearson omnibus normality test and the variance was tested using Bartlett’s test. For the normally distributed value, one-way ANOVA and Tukey’s *post hoc* test was used for statistical analysis. Otherwise, nonparametric Kruskal–Wallis test was used. The method of statistical analysis is specified in each figure legend.

## Code Availability

The MATLAB and ImageJ codes used for this study are available at GitHub: https://github.com/ThomasSchwarzLab/ArrayScanCodes; https://github.com/ThomasSchwarzLab/KymolyzerCodes; https://github.com/ThomasSchwarzLab/QuoVadoProCodes.

## Data Availability Statement

The datasets presented in this study can be found in online repositories. The names of the repository/repositories and accession number(s) can be found in the article/Supplementary Material.

## Ethics Statement

The animal study was reviewed and approved by the Institutional Animal Care and Use Committee (Boston Children’s Hospital).

## Author Contributions

HB, KH, and TS contributed to the design, supervised the study, organized the database, and wrote the manuscript. HB, AG, KH, and WW performed the experiments. HB and KH performed the statistical analysis. HB, KH, ES, and TS contributed to manuscript revision and reading. All authors approved the submitted version.

## Conflict of Interest

The authors declare that the research was conducted in the absence of any commercial or financial relationships that could be construed as a potential conflict of interest.

## Publisher’s Note

All claims expressed in this article are solely those of the authors and do not necessarily represent those of their affiliated organizations, or those of the publisher, the editors and the reviewers. Any product that may be evaluated in this article, or claim that may be made by its manufacturer, is not guaranteed or endorsed by the publisher.

## Supplementary Material

Supplementary Figure 1 Comparison of the effects of DMSO and calcimycin in the screening platform. **(A)** Representative fields with tracking of mitochondria as analyzed by the PATH algorithm in neurons treated with 10 μM DMSO or calcimycin. Stationary mitochondria are red and the tracks of moving mitochondria are blue. **(B)** From similar traces and using three concentrations of DMSO or 10 μM calcimycin, the mean of the integrated distance traveled and the KS2 *Z*-score are shown. Tukey’s box plot was used for all data, and the error bars represent SD.

Supplementary Figure 2 Mitochondrial motility in the presence of GSK-3β or CDKs inhibitors. The mean of integrated distance at three concentrations of six GSK-3β inhibitors **(A)** and six CDKs inhibitors **(B)**. Dotted lines represent the average value of mitochondria motility in DMSO treated neurons. More extensive dose-response curves over a broader concentrations range for dabrafenib **(C)**, rebastinib **(D)**, SU9516 **(E)**, tideglusib **(F)**, and TWS 119 **(G)** using at least six different concentrations. Tukey’s box plot was used for all data, and the error bars represent SD. All experiments were done in two independent cultures with four wells were imaged per condition, each containing dozens of axons.

Supplementary Table 1 Compounds screened. List of the 240 compounds and their sources whose screen results are summarized in Figure 1D. For each compound, the *Z*-scores of the median percent motile are given for each of two replicates. The values for the DMSO controls included on each plate are also shown.

## Abbreviations

PP2A: protein phosphatase 2A
GSK-3 β: glycogen synthase kinase 3 beta
CDK: cyclin-dependent kinase
PATHS: Particle Analysis and Tracking for High-throughput Screening
LAP: linear assignment problem
KS: Kolmogorov–Smirnov.
