# Supplementary figures and images for: Serine/Threonine Protein Phosphatase 2A Regulates the Transport of Axonal Mitochondria
Source: Front Cell Neurosci. 2022 Mar 18;16:852245. doi: 10.3389/fncel.2022.852245 (PMC8973303; doi:10.3389/fncel.2022.852245)

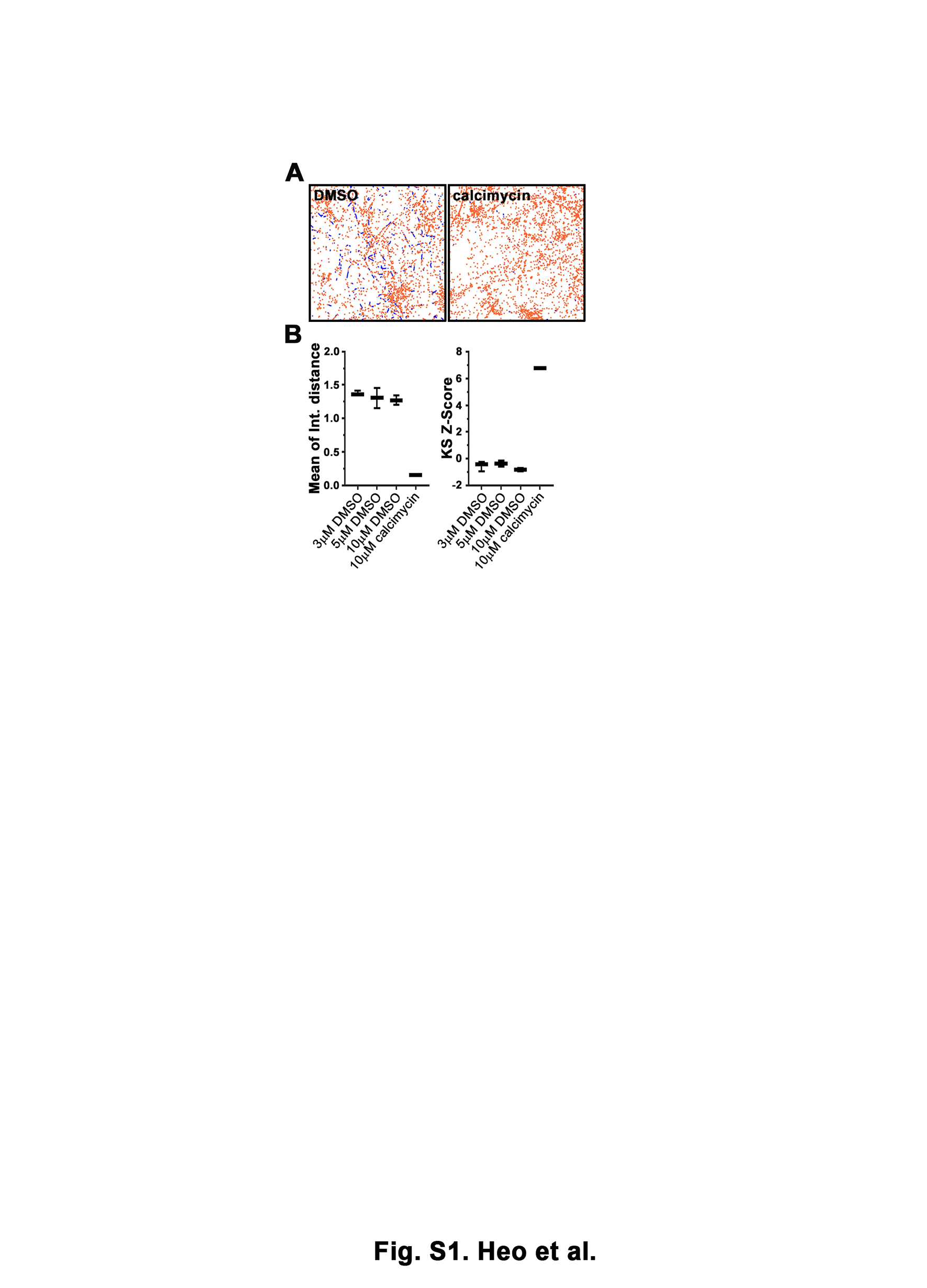

Supplement: Supplementary Figure 1 — Comparison of the effects of DMSO and calcimycin in the screening platform. (A) Representative fields with tracking of mitochondria as analyzed by the PATH algorithm in neurons treated with 10 μM DMSO or calcimycin. Stationary mitochondria are red and the tracks of moving mitochondria are blue. (B) From similar traces and using three concentrations of DMSO or 10 μM calcimycin, the mean of the integrated distance traveled and the KS2 Z-score are shown. Tukey’s box plot was used for all data, and the error bars represent SD. [file Image_1.TIF]

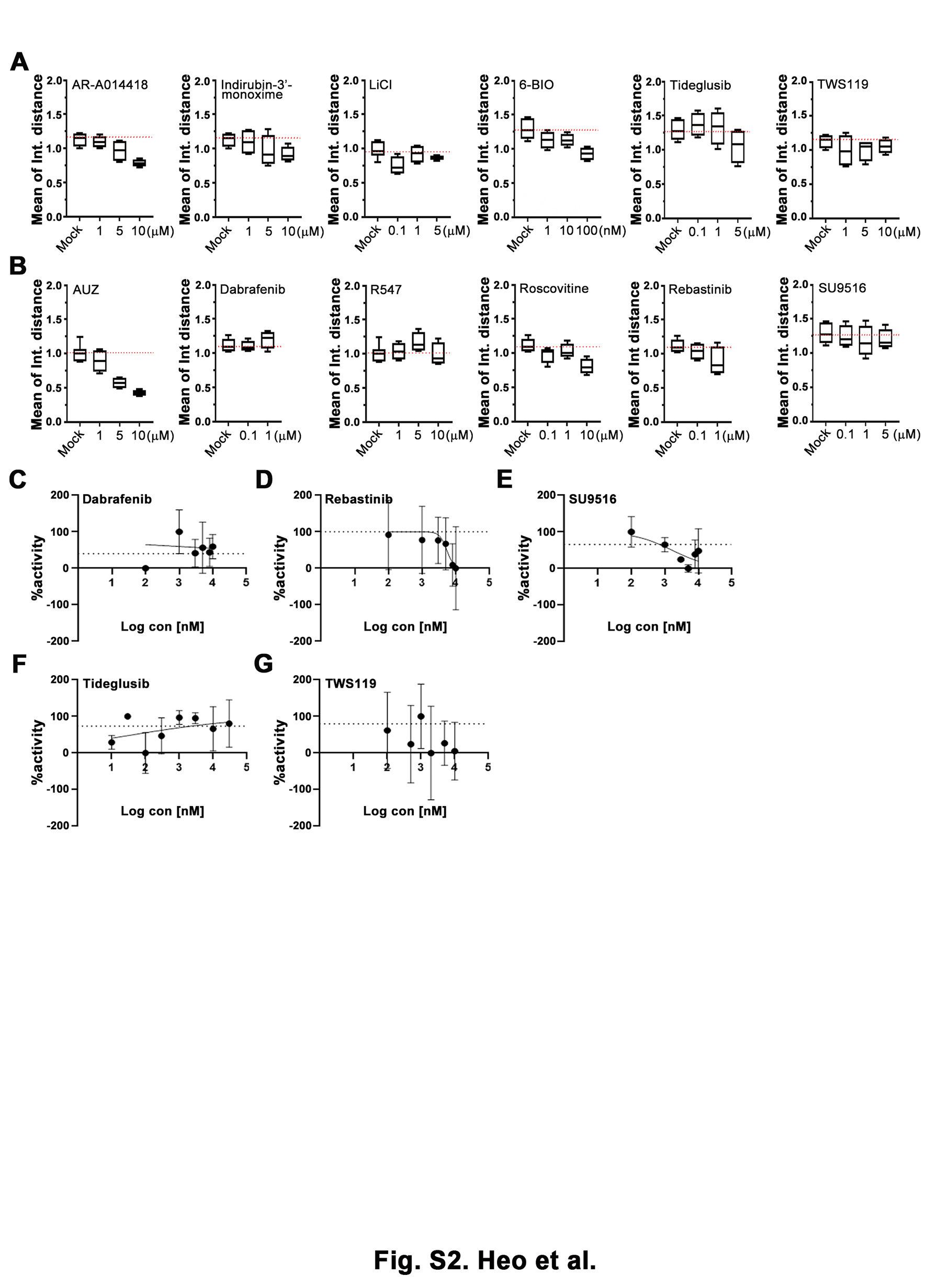

Supplement: Supplementary Figure 2 — Mitochondrial motility in the presence of GSK-3β or CDKs inhibitors. The mean of integrated distance at three concentrations of six GSK-3β inhibitors (A) and six CDKs inhibitors (B). Dotted lines represent the average value of mitochondria motility in DMSO treated neurons. More extensive dose-response curves over a broader concentrations range for dabrafenib (C), rebastinib (D), SU9516 (E), tideglusib (F), and TWS 119 (G) using at least six different concentrations. Tukey’s box plot was used for all data, and the error bars represent SD. All experiments were done in two independent cultures with four wells were imaged per condition, each containing dozens of axons. [file Image_2.TIF]
